# Effects of mycophenolate mofetil on kidney function and phosphorylation status of renal proteins in Alport COL4A3-deficient mice

**DOI:** 10.1186/s12953-014-0056-z

**Published:** 2014-12-10

**Authors:** Darinka Todorova Petrova, Frank Christian Schultze, Gunnar Brandhorst, Klaus-Dieter Luchs, Christof Lenz, Henning Urlaub, Diana Rubel, Oliver Gross, Philip D Walson, Michael Oellerich

**Affiliations:** Department of Clinical Pharmacology, Institute of Clinical Chemistry, University Medical Center Goettingen, Robert-Koch-Str. 40, 37099 Goettingen, Germany; Clinics of Gastroenterology and Endocrinology, University Medical Center Goettingen, Goettingen, Germany; Institute of Clinical Chemistry, University Medical Center Goettingen, Goettingen, Germany; Department of Clinical Pharmacology, University Medical Center Goettingen, Goettingen, Germany; Max Planck Institute for Biophysical Chemistry, Bioanalytical Mass Spectrometry Group, Goettingen, Germany; Clinics of Nephrology and Rheumatology, University Medical Center Goettingen, Goettingen, Germany

**Keywords:** Mycophenolic acid, Kidney fibrosis, Protein phosphorylation, Alport syndrome

## Abstract

**Background:**

We investigated the effects of mycophenolate mofetil (MMF) on kidney function and on protein phosphorylation in a mouse model for the human Alport syndrome.

**Methods:**

COL4A3-deficient (COL4A3−/−) mice were randomly allocated to receive a placebo (PLC COL4A3−/−) or MMF treatment (MMF COL4A3−/−). Wild type mice (WT) were used as controls. Changes in serum creatinine, total protein and blood urea nitrogen (BUN), concentrations of mycophenolic acid (MPA) and its glucuronide metabolite (MPAG), serum protein electrophoresis, urine dipstick chemistry and sediment were measured. Changes in the phosphorylation status of renal proteins and histology were analyzed.

**Results:**

MMF influenced kidney function and protein phosphorylation. Serum creatinine and BUN were lower in MMF treated compared to PLC treated COL4A3−/− mice. Serum albumin and alpha-1 globulins were significantly decreased while serum creatinine, alpha-2 globulins, urine dipstick protein, leukocyte esterase, hemoglobin and red blood cells were all increased in both COL4A3−/− groups compared to WT. Differential 2DE-gel analysis identified six phosphorylated kidney protein spots that were significantly altered by MMF.

**Conclusions:**

These data suggest that the MMF treatment in this murine model moderately improved kidney function and reversed the phosphorylation status of six renal phosphoprotein spots to that seen in WT mice.

**Electronic supplementary material:**

The online version of this article (doi:10.1186/s12953-014-0056-z) contains supplementary material, which is available to authorized users.

## Introduction

Mycophenolic acid (MPA) is the active metabolite of the pro-drug mycophenolate mofetil (MMF). It effectively and non-competitively inhibits inosine monophosphate dehydrogenase (IMPDH, EC 1.1.1.205). Human IMPDH is present in two isoforms: type I (expressed in nearly all cells) and type II (expressed in activated lymphocytes). IMPDH type II is about 5-fold more sensitive to MPA compared to IMPDH type I [1-3]. MPA inhibits the proliferation of T- and B-lymphocytes through the depletion of the nucleotides guanosine and deoxyguanosine thereby suppressing purine synthesis. It also inhibits the production of immunoglobulins [4]. MMF is one of the most commonly used immunosuppressive drugs either alone or in combination with other immunosuppressive drugs (e.g. corticosteroids and/or calcineurin inhibitors) for the prevention of organ rejection after solid organ transplantation as well as in the therapy of autoimmune and neoplastic diseases [3,5-8]. It is known that MPA, through a nonspecific mechanism of action, can also influence non-lymphatic cells such as fibroblasts [3,9-14]. It has been proposed that MPA also has a positive effect on the progression of human kidney fibrosis [15-17] based on numerous *in vivo* [9,17-22] and *in vitro* studies [10,12,17,23]. However, the mechanisms responsible for the effects of MMF on renal fibrosis, especially changes in the phosphoproteome, have not been adequately studied. The aim of the current study was to examine the effects of MMF treatment on kidney function and on the phosphorylation status of renal proteins in COL4A3-deficient (COL4A3−/−) mice, which represent an *in vivo*, non-hypertensive model for the autosomal form of Alport syndrome.

## Results

During the study period no severe MMF-related toxicity such as diarrhea was observed. However, animals were excluded from the study that became somnolent, exhibited a decrease in body weight of more than 20%, and/or had a total body weight of less than 16 g (n = 7). At the end of the 14 days of MMF treatment the COL4A3-deficient mice had lost less mean body weight than those treated with placebo (10% *vs*. 14%). The mean+/−SD body weight on the last day of the MMF treatment was 19+/−3 g in the MMF group, 18+/−1 g in the PLC group and 26+/−1 g in the wild-type control group (Additional file 1: Table S1A).

Two observers, independently and blinded to the treatment groups of COL4A3-deficient mice, examined the infiltration of mesangial matrix in 3653 areas after staining with hematoxylin and eosin. The tubulointerstitial fibrosis present after 2-weeks treatment of COL4A3−/− mice was only slightly inhibited by MMF in comparison to the PLC COL4A3 −/− mice as would be expected for this relatively short period of MMF treatment (WT: all areas with score = <1+; PLC: 13% - score 0, 34% - score 1+, 33% - score 2+, 20% - score 3+; MMF: 11% - score 0, 45% - score 1+, 31% - score 2+, 13% - score 3+), and glomerulosclerosis appeared to be unchanged by MMF (Additional file 2: Figure S1).

**Figure 1:**
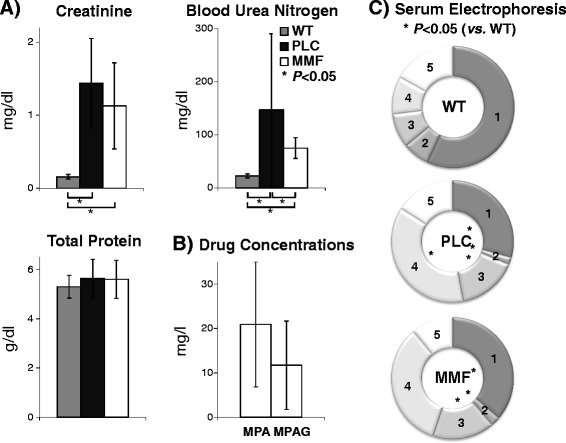
**Murine serum analysis using routine diagnostic methods. A)** *Clinical chemistry on an automated Roche system*: The concentrations of serum creatinine, blood urea nitrogen, and total protein are stratified for all three experimental groups (WT n = 3; PLC n = 10; MMF n = 10) and presented as the mean and standard deviation. **B)** *Drug concentrations*: The serum pre-dose concentrations of mycophenolic acid (MPA) and mycophenolic acid glucuronide (MPAG) are presented for the treatment group (MMF n = 10) as the mean and standard deviation. **C)** *Serum electrophoresis*: The first fraction (1) stands for albumin, second (2) - alpha-1 globulins, third (3) - alpha-2 globulins, forth (4) - beta globulins and the fifth (5) gamma globulins. The rings show the mean relative proportion of each separated fraction for all three experimental groups (WT n = 3; PLC n = 6; MMF n = 8). Legends: wild-type 129/SvJ mice (WT); placebo COL4A3−/− mice (PLC); COL4A3−/− mice treated with 100 mg/kg mycophenolate mofetil per day (MMF); *P* < 0.05 using the Mann–Whitney-*U* test (*).

Clinical chemistry protocols for routine measurements in human samples are applicable even for the small murine sample volumes used. Serum and urine biochemical parameters are shown in Figure 1 and in Additional file 1: Table S1. Serum creatinine concentrations were significantly increased (*P* < 0.05; Figure 1A; Additional file 1: Table S1B) in both COL4A3-deficient groups compared to WT mice (WT: 0.16+/−0.03; PLC: 1.44+/−0.61; MMF: 1.13+/−0.59 mg/dl). Total protein did not show any significant differences (*P* > 0.05; Figure 1A; Additional file 1: Table S1B) between the three experimental groups (WT: 5.30+/−0.47; PLC: 5.64+/−0.78; MMF: 5.61+/−0.77 g/dl) but blood urea nitrogen was significantly lower (*P* < 0.05; Figure 1A; Additional file 1: Table S1B) in the WT group compared to both COL4A3-defitient groups (WT: 22.47+/−3.97; PLC: 147.00+/−143.61; MMF: 74.75+/−19.67 mg/dl).

Pre-dose MPA and mycophenolic acid glucuronide (MPAG) serum concentrations were below the lower limit of quantification (<0.5 mg/l MPA and 5 mg/l MPAG for 1:5 diluted samples) in the WT and PLC groups. However, MPA concentrations were between 20 and 41 mg/L (mean+/−SD = 21+/−14) and MPAG concentrations were between 9 and 26 mg/l (12+/−10) in the MMF group of COL4A3−/− mice (Figure 1B; Additional file 1: Table S1C).

Results of the serum electrophoresis (presented in Figure 1C and in Additional file 1: Table S1D) revealed a significant decrease (*P* < 0.05) in albumin (WT: 3.02+/−0.26; PLC: 1.72+/−0.44; MMF: 2.10+/−0.53 g/dl) and alpha-1 fractions (WT: 0.37+/−0.04; PLC: 0.11+/−0.05; MMF: 0.15+/−0.07 g/dl) in both COL4A3-deficient groups but a significant increase (*P* < 0.05) in the alpha-2 fractions (WT: 0.48+/−0.04; PLC: 0.93+/−0.30; MMF: 0.95+/−0.28 g/dl) but no significant difference (*P* > 0.05) in the beta fraction (WT: 0.53+/−0.13; PLC: 2.18+/−1.06; MMF: 1.95+/−1.23 g/dl) or gamma fraction (WT: 0.89+/−0.07; PLC: 0.94+/−0.49; MMF: 0.64+/−0.33 g/dl).

Urine chemistry (Additional file 1: Table S1E) demonstrated increases in total urine protein in both COL4A3-deficient groups (MMF & PLC: 100–300 mg/dl; WT: < 15 mg/dl). Hemoglobin and leucocyte esterase were consistently negative in the WT group in contrast to both COL4A3-deficient groups (hemoglobin: ca. 100 mg/dl; leucocyte esterase: ca. 250 leucocytes/μl). None of the other urine chemistry results showed any significant differences (glucose, bilirubin, urobilinogen, nitrite, ketones, pH, and specific gravity). The urine sediment also showed differences between experimental groups (Additional file 1: Table S1F). Erythrocytes were observed microscopically only in urine samples from both COL4A3-deficient groups and these mice also had less bacteria and amorphous phosphates than was seen in the WT group. In addition, leucocytes were detected in urine from WT and MMF-treated COL4A3−/− mice as well as yeasts and occasionally hyaline cylinders were found in samples from WT and PLC COL4A3−/− mice.

**Figure 2:**
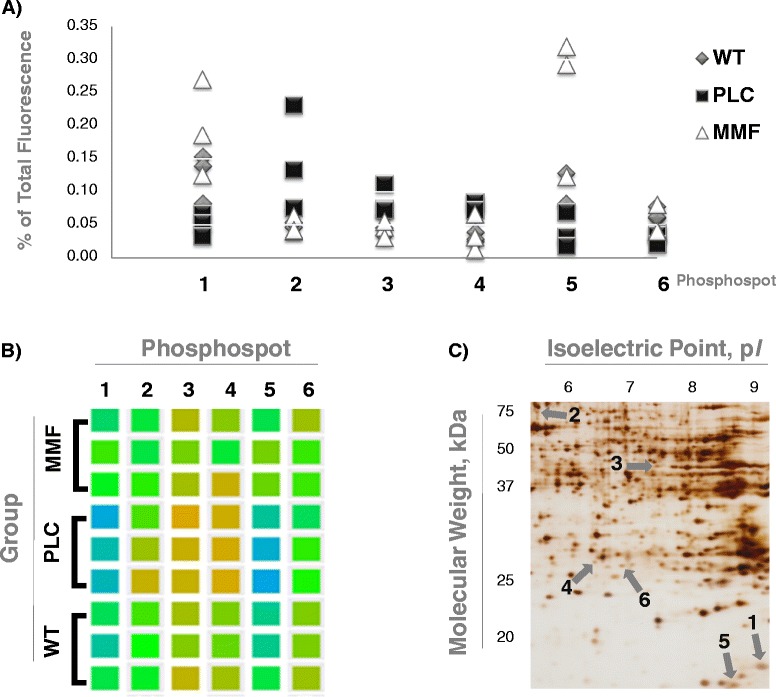
**Differentially phosphorylated protein spots in fibrotic kidneys reversed by mycophenolate mofetil. A)** *Fluorescence*: The point diagram presents the percentage of the relative fluorescence of the whole gel (100%), as well as stratification for all three experimental groups (WT n = 3 in gray; PLC n = 3 in black; MMF n = 3 in white). **B)** *Cluster check*: The hit phosphospots are shown as squares in different colors, depending on the fluorescence in the original scans of the phospho-stained 2DE gels (using the analysis tool of the Delta2D software). The stratification is done for all three experimental groups (WT n = 3; PLC n = 3; MMF n = 3). **C)** *Silver-stained 2DE gel*: The picture visualizes the phosphospots of one representative WT mouse, as well as the isoelectric point (p*I*) and the molecular weight (kDa) in this zoomed-in area. Legends: wild-type 129/SvJ mice (WT); placebo treated COL4A3−/− mice (PLC); and COL4A3−/− mice treated with 100 mg/kg mycophenolate mofetil per day (MMF).

Differential 2-DE analysis of kidney proteins using a phosphorylation-specific stain revealed six protein spots (out of ca. 500) that exhibited significant phosphorylation changes in WT vs. PLC and MMF vs. PLC (*P* < 0.05) groups. There were no significant differences; however, between WT and MMF groups (*P* > 0.05; Figure 2; Additional file 1: Table S1G). Three differentially phosphorylated protein spots (numbers 1, 5 and 6) were down-regulated in PLC COL4A3−/− mice, while another three spots (numbers 2, 3 and 4) were up-regulated. MMF treatment reversed the phosphorylation of all six spots to match that seen in WT mice (Figure 2). These spots were identified in duplicate samples using mass spectrometry from three replicate 2DE-gels each from one representative WT, one PCL and one MMF mouse. Identified proteins were considered only if the total unique peptide count was > = 3. For all replicates, comparable sets of protein species were identified (see Additional file 3: Table S2 for a list of proteins derived from the WT mice). Even after removal of laboratory contaminants such as human keratin, all of the 2-DE spots examined contained more than one protein, indicating a potential overlay with neighboring spots. From the results lists, known phosphoproteins were selected as potential markers and checked for plausibility by comparing the observed molecular weight and isoelectric point to the expected values (Table 1).

**Table 1:** **Potential novel biomarkers for Alport syndrome**

**Phosphospot**	**Phosphoprotein identified in the spot**	**Quantitative value (Normalized total spectra)**	**Molecular weight, kDa**		**Isoelectric point, p** ***I***		
	**(Entry Name)**	**WT/PLC/MMF**	**Observed**	**Calculated**	**Observed**	**Basal, unphosphorylated state** ^**a**^	**Prediction for various phosphorylation states** ^**a**^
**Phosphospot 1**			17		9.0		
	Actin-related protein 2/3 complex subunit 3 (ARPC3_MOUSE)	9/13/53		21		8.78	1-18 phosphosites; 8.28-4.09
Main protein	NADH dehydrogenase [ubiquinone] 1 alpha subcomplex subunit 8 (NDUA8_MOUSE)^b^	35/49/53		20		8.76	1-11 phosphosites; 8.41-5.45
**Phosphospot 2**			75		5.5		
	Plastin-3 (PLST_MOUSE)	2/0/0		71		5.42	1-44 phosphosites; 5.36-4.19
	Heat shock cognate 71 kDa protein (HSP7C_MOUSE)	2/0/0		71		5.37^c^	1-137 phosphosites; 5.32-2.38^c^
	Adseverin (ADSV_MOUSE)	5/18/58		80		5.64	1-17 phosphosites; 5.57-4.93
Main protein	Serum albumin (ALBU_MOUSE)	33/13/99		69		5.75	1-120 phosphosites; 5.69-2.63
**Phosphospot 3**			45		7.5		
	ATP synthase subunit alpha, mitochondrial (ATPA_MOUSE)	16/0/0		60		9.22	1-79 phosphosites; 9.07-3.10
	Catalase (CATA_MOUSE)	2/0/0		60		7.72	1-69 phosphosites; 7.19-3.73
Main protein	Glutamate dehydrogenase 1, mitochondrial (DHE3_MOUSE)	43/22/37		61		8.05	1-82 phosphosites; 7.40-3.33
**Phosphospot 4**			27		6.5		
	Serine/arginine-rich splicing factor 1 (SRSF1_MOUSE)	4/0/0		28		10.37	1-58 phosphosites; 10.22-3.08
	26S proteasome non-ATPase regulatory subunit 9 (PSMD9_MOUSE)	4/0/0		25		6.00	1-14 phosphosites; 5.83-4.58
Main protein	Estradiol 17-beta-dehydrogenase 8 (DHB8_MOUSE)^b^	9/30/17		27		6.10	1-5 phosphoprotein; 5.81-5.07
**Phosphospot 5**			15		8.5		
	Destrin OS (DEST_MOUSE)	15/41/10		19		8.14	1-30 phosphosites; 7.15-3.20
	40S ribosomal protein S14 (RS14_MOUSE)	5/0/56		16		10.07	1-15 phosphosites; 9.84-5.26
Main protein	Peptidyl-prolyl cis-trans isomerase A (PPIA_MOUSE)	72/30/44		18		7.73	1-39 phosphosites; 6.75-2.36
**Phosphospot 6**			26		7.0		
	Serine/arginine-rich splicing factor 1 (SRSF1_MOUSE)	12/1/27		28		10.37	1-58 phosphosites; 10.22-3.08
Main protein	Omega-amidase NIT2 (NIT2_MOUSE)^b^	49/29/34		31		6.44	1-13 phosphosites; 5.10-4.58

^a^ http://www.phosphosite.org; January 2014.

^b^Main protein identified in each group, not yet described as phosphoprotein.

^c^Information for the human protein; March 2014.

The table presents possible phosphoproteins, including the main proteins identified in this mouse model for the human Alport syndrome.

## Discussion

Alport glomerulonephritis is a hereditary disorder leading to kidney fibrosis that is associated with mutations in genes encoding for COL4A3, COL4A4 or COL4A5, a gene necessary for the normal structure of the glomerular basement membrane. If these genes are mutated, the glomerular basement membrane will be altered [24]. In this *in vivo* study, COL4A3−/− mice served as a model for progressive renal disease seen in the human Alport syndrome [25]. Untreated COL4A3 −/− mice die from renal failure typically after 66 to 71 days [22,26-28], whereas the normal life span of WT mice is 565 days [27]. COL4A3−/− mice have previously been used to study the nephroprotective and antifibrotic effects of different drugs. The life span has been reported to be prolonged by 13% after treatment with paricalcitol [29], by 19% after treatment with etanercept [26], by 25% after treatment with BX471 [30], by 28% after treatment with cerivastatin [28], by >50% after treatment with ramipril [29], by >68% after combined treatment with paricalcitol and ramipril [29], or even by >100% after treatment with ramipril [27]. Previously, we were able to demonstrate improved kidney function in MMF-treated COL4A3−/− mice although the overall survival was not improved [22] and we therefore suggested that, in contrast to the other drugs studied, MMF might have an inhibitory effect on the initial tubulointerstitial fibrosis but not on glomerulosclerosis. The proteome changes we found supported this suggestion [31]. To explore the cause of these contradictory findings further, we investigated the effects of MMF on renal function with a special focus on screening for phosphoproteomic differences using total protein extracts from the kidneys of 7-week old male WT, PLC treated COL4A3−/− and MMF-treated COL4A3−/− mice.

Pre-dose serum MPA and MPAG concentrations (Figure 1B, Additional file 1: Table S1C) both showed inter-individual variability as previously reported [22]. The mean MPA concentration was ca. 21 mg/l and the mean MPA and MPAG concentrations were ca. 12 mg/l without any signs of toxicity in these experimental mice as has previously been reported after treatment with 10, 50, 100 and 150 mg MMF/kg/day [22]. Interestingly, MPA and MPAG concentrations in the COL4A3−/− mice were much higher than seen in patients following solid organ transplantation. A preliminary therapeutic range for pre-dose MPA concentrations in renal transplantation patients during the first 3 months post-surgery (when used in association with cyclosporine) was only 1.0 to 3.5 mg/l [32]. In a previous study, female Wistar rats aged 12 weeks were treated with 20, 30 or 40 mg/kg MMF once daily using gastric feeding tubes and the group receiving the highest dose of 40 mg/kg developed diarrhea after 26–28 days of treatment [33]. This adverse effect was neither seen in our previous study [22], nor in the present study with COL4A3−/− mice suggesting a higher tolerance for MMF in our male mouse model as compared to female Wistar rats.

Serum creatinine, total protein, as well as the 5 serum electrophoresis fractions did not differ significantly between PLC COL4A3−/− and MMF treated COL4A3−/− mice (Figure 1, Additional file 1: Table S1): results which are consistent with the reported moderate effect of this immunosuppressive drug treatment on survival [22]. However, the blood urea nitrogen level after 2-weeks treatment with MMF was significantly decreased, consistent with our previous report [22] and thus suggesting again that tubulointerstitial fibrosis can be inhibited by MMF [31]. Albumin and alpha-1 globulins were decreased while creatinine, alpha-2 globulins, urine dipstick protein, leukocyte esterase (leukocytes), hemoglobin and red blood cells were all increased in both COL4A3−/− groups compared to WT, thereby confirming the presence of glomerulosclerosis.

We found 6 differentially phosphorylated spots (Figure 2). The database search included serine, threonine and tyrosine phosphorylation as variable peptide modifications, however no phosphorylated peptides were identified at sufficient confidence. This is likely due to the substoichiometric nature of protein phosphorylation. Due to the already low abundance of the protein spots, phosphopeptide enrichment strategies were not pursued as they usually require sufficient starting amounts. Protein identifications are summarized in Additional file 1: Table S1 G and Table 1.

The differentially phosphorylated protein spot 1 (observed 17 kDa/9 p*I*) was significantly down-regulated in PLC COL4A3−/− mice. MMF treatment of the COL4A3−/− mice increased the phosphorylation to match that of the WT mice. The major protein in the spots of all three groups was NADH dehydrogenase [ubiquinone] 1 alpha subcomplex subunit 8 (NDUA8), however NDUA8 is not described as a phosphoprotein. The spot also contained one possible phosphoprotein: actin-related protein 2/3 complex subunit 3 (ARPC3), an actin-binding cytoskeleton protein that is involved in cell projection and lamellipodium and functions as a component of the Arp2/3 complex. The Arp2/3 complex generates branched actin filaments in motile cells that drive the cell front forward [34]. In a recent study it was reported that threonine and tyrosine phosphorylation are important for a subset of the functions of the Arp2/3 complex, including the regulation of development [35].

The phosphorylation of phosphospot 2 (observed 75 kDa/5.5 p*I*) was significantly up-regulated in PLC COL4A3−/− mice, while the MMF treatment of the COL4A3−/− mice reversed the phosphorylation to be similar to that seen in WT mice. This spot contained four proteins previously described as phosphoproteins: serum albumin (ALBU), adseverin (ADSV), heat shock cognate 71 kDa protein (HSP7C) and plastin-3 (PLST). ALBU was the most abundant protein in the spot as indicated by spectral counts. Phosphorylation of ALBU was also observed in the extracellular medium. This protein is involved in the regulation of the colloidal osmotic pressure of blood, transport, cellular responses to starvation, negative regulation of apoptosis, and other processes. The second possible phosphoprotein was ADSV, involved in the actin filament capping, the negative regulation of cell proliferation, as well as in the positive regulation of apoptosis. HSP7C and PLST were identified at low abundance: HSP7C inhibits the transcriptional coactivator activity of CITED1 on Smad-mediated transcription, whereas PLST, the actin-binding protein in intestinal microvilli and fibroblast filopodia, is involved in motility, polarity and chemotaxis. Interestingly, PLST belongs to the plastin protein family of three isoforms with relatively high homology between: I (plastin-1 expressed in the intestine and kidney), L (plastin-2 in leukocytes and cancer) and T (plastin-3 in solid tissues) [36]. Only L-plastin has been reported (to date) to be phosphorylated [37] and it has been identified as a transformation-induced polypeptide of neoplastic fibroblasts [36]. Moreover, L-plastin is included in a novel serum triple marker assay for the early detection of malignant kidney tumors [38].

While the phosphospot 3 (observed 45 kDa/7.5 p*I*) showed a significant increase in phosphorylation in PLC COL4A3−/− mice, MMF treatment of the COL4A3−/− mice reversed this phosphorylation. This spot matched three possible phosphoproteins: the main protein was glutamate dehydrogenase 1 (DHE3), but ATP synthase subunit alpha (ATPA) (involved in the embryonic development and negative regulation of endothelial cell proliferation), and catalase (CATA) were also identified. Although identified at low spectral count, indicating low relative abundance in the spot, CATA is still of high interest with respect to kidney fibrosis. CATA is the peroxisome enzyme, oxidoreductase, involved in numerous biological processes including kidney development, negative regulation of apoptosis positive regulation of cell division, and a selenium-centered micronutrient biological network. Interestingly, CATA promotes growth of a number of cell types including T-cells, B-cells, and both normal and transformed fibroblast cells. One recent study showed that T lymphocytes and IL-6 play important roles in renal fibrosis [39]. Another group has reported that monocytes may influence myofibroblast accumulation via TGF-beta 1, and that monocytes, but not myofibroblasts, are associated with tubular atrophy in Alport mice [40].

The phosphoprotein spot 4 (observed 27 kDa/6.5 p*I*) showed significantly increased phosphorylation in PLC COL4A3−/− mice and MMF treatment of the COL4A3−/− mice reversed this to become similar to that seen in WT mice. This spot matched two possible phosphoproteins: serine/arginine-rich splicing factor 1 (SRSF1) and the 26S proteasome non-ATPase regulatory subunit 9 (PSMD9). The most abundant protein identified in all three groups has not yet been described as a phosphoprotein: estradiol 17-beta-dehydrogenase 8 (DHB8).

The phosphoprotein spot 6 (observed 26 kDa/7 p*I*) was significantly down-regulated in PLC COL4A3−/− mice, but MMF treatment of the COL4A3−/− mice increased the phosphorylation to that seen in WT mice. In all three groups this spot contained one main protein, omega-amidase NIT2 (NIT2), not previously described as a phosphoprotein, and one possible phosphoprotein: the above mentioned SRSF1. We postulate that the increased phosphorylation of phosphospot 4 and the decreased phosphorylation of phosphospot 6 are related to SRSF1 because it is known that increasing phosphorylation of the phosphosites can shift the p*I* to lower values (Phosphospot 4, 6.5 p*I* observed, = 18 phosphorylated phosphosites vs. Phosphospot 6, 7 p*I* observed, = 14 phosphorylated phosphosites, Figure 2C). SRSF1 is involved in the regulation of constitutive and alternative splicing. In a recent review it was noted that SRSF1 has also been shown to promote tumor transformation and growth by several mechanisms; for example, by stabilizing mRNA of anti-apoptotic factors 21 and by generating inactive tumor suppressor proteins by alternative splicing [41]. Reversible phosphorylation cascades are able to rapidly conduct signals throughout the cell and are probably important in mediating extracellular signals to the spliceosome [42].

The differentially phosphorylated protein spot 5 (observed 15 kDa/8.5 p*I*) was significantly down-regulated in PLC COL4A3−/− mice and MMF treatment of the COL4A3−/− mice reversed this down regulation. This spot contained three possible phosphoproteins: peptidyl-prolyl cis-trans isomerase (PPIA), 40S ribosomal protein S14 (RS14) and destrin (DEST). PPIA, which appears to be most abundant phosphoprotein in the spot, is involved in inflammation, acceleration of the folding of proteins, and in the positive regulation of protein secretion. In a recent study using human colon cancer cells, DEST appeared to be required for cell migration and invasion in response to a pro-invasive neuroendocrine peptide. This property was related to a DEST-dependent phosphorylation of a p130Crk-associated substrate (p130Cas) upon cell adhesion [43]. The structural constituent of ribosome RS14 (regulation of translation) however was only present in very low quantity in the spot as indicated by spectral counts.

## Conclusions

The current data confirmed the ability of MMF to moderately improve kidney function in a mouse model of human Alport syndrome, presumably through inhibition of tubulointerstitial fibrosis. MMF reversed the COL4A3 related phosphorylation status of renal proteins in this murine fibrotic kidney model to that seen in WT mice. The involved phosphoproteins are associated with a number of important cell properties including: cell projection, motility, migration, invasion, polarity, division, transformation, and cell growth (T-cells, B-cells, fibroblasts). These results support our *in vitro* findings using functional assays with the human epithelial HK-2 cell line from proximal tubuli [44] and on the monkey renal fibroblast COS-7 cell line [13]. It may be speculated that the MMF treatment was initiated too late or the duration was too short to reverse the changes leading to renal fibrosis in the treated COL4A3−/− mice. Further studies are necessary to clarify, validate or extend these preliminary findings.

## Methods

### COL4A3-deficient mice

This study was approved by the local German authorities (LAVES, Oldenburg, Germany, 2011) and supervised by veterinarians. COL4A3−/− mice were obtained from Jackson Laboratory (Bar Harbor, ME). They were bred on a 129/SvJ genetic background, to reduce individual differences, under pathogen-free housing conditions with a 12-hour dark and light period and unlimited access to food and water at the local animal facility. DNA was isolated using the DNeasy kit® (Blood & Tissue Kit; QIAGEN GmbH, Hilden, Germany). Genotyping of the murine *COL4A3* gene was conducted according to Cosgrove et al. [24]. Twenty 5-week old COL4A3−/− mice, all male in order to avoid gender-specific differences, and with a body weight of more than 16 g, were randomly allocated to two experimental groups (PLC: n = 10, MMF: n = 10). They were treated daily by gavage with either an MMF solution (100 mg/kg) or with an equivalent amount of vehicle placebo (PLC) as described previously [22]. They were examined daily and their body weight was documented. After 14 days of treatment they were sacrificed using an approved protocol. An additional three wild type mice were used as controls (WT: mouse strain 129/SvJ), because reference values for the parameters studied in the WT mouse strain were not available. Serum, urine, and kidney tissues were collected 24 hours after the last MMF treatment and stored at −80°C for further analysis. All results obtained in serum from COL4A3-deficient groups were corrected with a dilution factor (1:5 or 1:10 dilution; *vol*/*vol* using 0.9% NaCl). Serum from WT mice was not diluted except for serum electrophoresis which was diluted 1:10 in all three groups.

### Histological staining with hematoxylin and eosin

Kidney tissue sections (paraffin-embedded) were prepared from each mouse and histological evaluation was performed after staining with hematoxylin and eosin by two observers blinded to the groups as described previously [31]. Tubulointerstitial fibrosis was scored semi-quantitatively using light microscopy in accordance with the Banff criteria [45]: 0 (no tubulointerstitial changes); 1+ (less than 25%); 2+ (25–50%) and 3+ (more than 50%). Additionally, histological evaluation and protein isolation were also done on serial cryo-slides (Microtome, Microm HM325; Thermo Scientific, Walldorf, Germany).

### Clinical chemistry: serum and urine

Serum concentrations of creatinine, blood urea nitrogen, and total protein were quantified using a Cobas 8000 modular analyzer (Roche Diagnostics GmbH, Mannheim, Germany) as described elsewhere [22] using pre-diluted samples (1:5). Serum electrophoresis was also conducted using pre-diluted samples (1:10) and routine diagnostic equipment (Hyrys 2, Sebia GmbH, Fulda, Germany).

Urine dipstick chemistries and microscopic examinations were performed as follows: 3–10 μl sample per analyte were analyzed on 11-field-AUTION dipsticks (URIFLET S; ARKRAY Factory Inc, Shiga, Japan) using an AUTION analyzer (AUTION MAX; ARKRAY Factory Inc, Shiga, Japan) and microscopic evaluations of the urine sediments were done using a phase-contrast microscope (Zeiss Standard 14 with objective 5165602 and ocular K-pl-W 12,5x/18; Carl Zeiss Microscopy GmbH, Jena, Germany; 400-fold total magnification) on 100 μl samples after centrifugation (2 min at ca. 3000 × g; centrifuge type 3530; Abbott Laboratories GmbH, Hannover, Germany).

### Drug concentrations

Pre-dose serum concentrations of mycophenolic acid (MPA) and mycophenolic acid glucuronide (MPAG) were measured in pre-diluted samples (1:5) using a Quattro Premier XE Triple Quadrupole mass spectrometer (Waters Corporation, Milford Massachusetts, U.S.A.) as previously described [22].

### Two-dimensional electrophoresis (2DE)

Cryo-slides of kidneys (3 mice per group: WT, PCL, and MMF) were used for protein isolation as previously described [31]. Briefly, protein concentrations were determined with the Bradford method [46]. Two-dimensional electrophoresis (2-DE) gels each with 125 μg protein/immobilized pH gradient strip (17-cm; nonlinear pH range of 3–10; ReadyStrip TM; Bio-Rad) were produced using the method of Görg et al. [47]. The strips were passively rehydrated and the proteins were then focused in a Protein IEF Cell (Bio-Rad). The strips were loaded onto a vertical 12.5% polyacrylamide gel for further separation. The gels with spots separated according to both isoelectric point and molecular weight were stained first with Ready Solution Pro-Q® Diamond Phosphoprotein Gel Stain according to the manufacturer’s recommendations (Invitrogen, Ltd., Paisley, UK) to produce fluorescence of differentially phosphorylated proteins as previously described [48]. Subsequently, scans were done using a fluorescence scanner (FLA 5100; Fujifilm Europa GmbH, Düsseldorf, Germany). In order to visualize and quantify the protein spots, the gels were further stained with silver according the modified method of Blum et al. [49]. Scans of the phospho-stained gels and silver-stained gels were densitometrically quantified using the Delta2D software (Version 4.2, DECODON GmbH, Greifswald, Germany). The spot intensity was evaluated as a percentage of the total intensity in each gel. A statistical tool integrated into the Delta2D software was used to evaluate the differences in protein phosphorylation between the groups. Protein spots of interest were those spots that differed significantly between WT and PLC COL4A3−/− mice but without any statistically significant difference between WT and MMF COL4A3−/− mice. A change of at least 50% in phosphorylation was used as the cut off for spot selection. Selected spots were excised and digested in-gel with trypsin according to Shevchenko et al. [50].

### Protein identification by mass spectrometry

Mass spectrometric analysis was carried out with peptide mixtures after preconcentration on a Reversed Phase-C18 precolumn (0.15 mm ID × 20 mm self-packed with Reprosil-Pur120 C18-AQ 5 μm, Dr. Maisch, Ammerbuch-Entringen, Germany) and separation by Reversed Phase-C18 nanoflow chromatography [0.075 mm ID × 200 mm Picofrit column (New Objective, Woburn/MA, USA) self-packed with Reprosil-Pur 120 C18-AQ, 5 μm] using a 15 min linear gradient on a nanoLC 425 nanoflow chromatography system (AB SCIEX, Framingham/MA, U.S.A.). The eluent was analyzed using a Top10 method in the Data Dependent Acquisition mode on a TripleToF 5600+ QqToF mass spectrometry system operated using Analyst TF1.6 software (AB SCIEX, Foster City/CA, USA). For database searching, tandem mass spectra were extracted using AB SCIEX MS Data Converter software v1.3 beta. All MS/MS samples were analyzed using Mascot v2.4.1 software (Matrix Science, London, UK) set up to search the UniProt/SwissProt database (release 02/14 filtered for *Mus musculus*, 16665 entries) with mass tolerances of 10 ppm for precursors and 0.05 Da for fragments, respectively. Cysteine carbamidomethylation was used as a fixed modification, methionine oxidation and serine, threonine and tyrosine phosphorylation as the variable modification. Scaffold v4.0.5 software (Proteome Software Inc., Portland/OR, USA) was used to validate MS/MS based peptide and protein identifications. Peptide identifications were accepted if they could be established at greater than 95.0% confidence. Protein identifications required a minimum of three confident peptide identifications and a protein confidence threshold of 99.0%. Spectral counts (i.e. the number of MS/MS sequencing events leading to identification of the same protein) were used to estimate the relative abundance of proteins contained in a single spot. From the results list, known phosphoproteins were selected using publicly available on-line resources (http://www.phosphosite.org and http://www.genecards.org, January 2014).

### Statistics

The significance of the differences between the experimental groups was calculated using the Mann–Whitney *U* test (IBM SPSS 20, Ehningen, Germany). *P* < 0.05 was considered statistically significant.

## Additional files

Additional file 1: Table S1. Data Overview. The information derived from the experimental mice (A: Body weight of mice at 7 weeks of age), serum (B: Clinical chemistry; C: Drug concentrations; D: Serum protein electrophoresis) and urine samples (E: Urine dipstick; F: Urine sediment) as well as kidney tissues (G: Differentially phosphorylated protein spots) are presented as the mean and standard deviation along with *P*-value according to Mann–Whitney-*U* test if appropriate. Additional file 2: Figure S1. H & E staining. The pictures illustrate the histochemical data from one representative wild-type 129/SvJ mouse (WT); one placebo treated COL4A3−/− mouse (PLC); and one COL4A3−/− mouse treated with 100 mg/kg mycophenolate mofetil per day (MMF). Additional file 3: Table S2. Protein Overview. The matrix presents an unfiltered list of all proteins identified from the total unique peptides (more than 3) in the six phosphospots of interest. The protein identification presented here was done in one representative wild-type mouse. The main spot protein (printed in bold) was the same as in the PLC and MMF COL4A3−/− mice.

## Abbreviations

IMPDH: Inosine monophosphate dehydrogenase
MPA: Mycophenolic acid
MMF: Mycophenolate mofetil
MPAG: Mycophenolic acid glucuronide
PLC: Placebo
2-DE: Two-dimensional electrophoresis
WT: Wild type
