# Supplementary material for: Effects of mycophenolate mofetil on kidney function and phosphorylation status of renal proteins in Alport COL4A3-deficient mice
Source: Proteome Sci. 2014 Dec 10;12:56. doi: 10.1186/s12953-014-0056-z (PMC4269973; doi:10.1186/s12953-014-0056-z)
Supplement: Additional file 1: Table S1. — Data Overview. The information derived from the experimental mice (A: Body weight of mice at 7 weeks of age), serum (B: Clinical chemistry; C: Drug concentrations; D: Serum protein electrophoresis) and urine samples (E: Urine dipstick; F: Urine sediment) as well as kidney tissues (G: Differentially phosphorylated protein spots) are presented as the mean and standard deviation along with P-value according to Mann–Whitney-U test if appropriate. [file 12953_2014_56_MOESM1_ESM.docx]

| **Parameter** | **Group** | **N** | **Unit** | **Mean value** | **Standard Deviation** |  | ***P*-value** |
| --- | --- | --- | --- | --- | --- | --- | --- |
| ***A: Body weight of mice at 7 weeks of age*** | | |  |  |  |  |  |
|  | WT | 3 | g | 26.367 | 0.850 | **WT *vs.* PLC** | ***P*=0.011** |
|  | PLC | 10 | g | 18.480 | 1.333 | PLC *vs.* MMF | *P*=0.821 |
|  | MMF | 10 | g | 19.210 | 3.232 | **WT *vs.* MMF** | ***P*=0.018** |
| ***B: Clinical chemistry, Serum parameters*** | | |  |  |  |  |  |
| **Creatinine** |  |  |  |  |  |  |  |
|  | WT | 3 | mg/dl | 0.157 | 0.031 | **WT *vs.* PLC** | ***P*=0.011** |
|  | PLC | 10 | mg/dl | 1.440 | 0.609 | PLC *vs.* MMF | *P*=0.364 |
|  | MMF | 10 | mg/dl | 1.130 | 0.589 | **WT *vs.* MMF** | ***P*=0.043** |
| **Total Protein** |  |  |  |  |  |  |  |
|  | WT | 3 | g/dl | 5.300 | 0.465 | WT *vs.* PLC | *P*=0.397 |
|  | PLC | 10 | g/dl | 5.640 | 0.776 | PLC *vs.* MMF | *P*=0.940 |
|  | MMF | 10 | g/dl | 5.605 | 0.773 | WT *vs.* MMF | *P*=0.612 |
| **Blood Urea Nitrogen** |  |  |  |  |  |  |  |
|  | WT | 3 | mg/dl | 22.467 | 3.972 | **WT *vs.* PLC** | ***P*=0.011** |
|  | PLC | 10 | mg/dl | 147.000 | 143.609 | **PLC *vs.* MMF** | ***P*=0.037** |
|  | MMF | 10 | mg/dl | 74.750 | 19.673 | **WT *vs.* MMF** | ***P*=0.011** |
| ***C: Drug Concentrations, Serum parameters*** | | | |  |  |  |  |
| **Mycophenolic Acid** |  |  |  |  |  |  |  |
|  | WT | 3 | mg/l | <0.1^&^ | 0 |  |  |
|  | PLC | 10 | mg/l | <0.5^§^ | 0 | **PLC *vs.* MMF** | ***P*=<0.001** |
|  | MMF | 10 | mg/l | 20.930 | 14.111 | **WT *vs.* MMF** | ***P*=0.011** |
| **Mycophenolic Acid Glucuronide** | | |  |  |  |  |  |
|  | WT | 3 | mg/l | <1^&^ | 0 |  |  |
|  | PLC | 10 | mg/l | <5^§^ | 0 | **PLC *vs.* MMF** | ***P*=0.002** |
|  | MMF | 10 | mg/l | 11.710 | 9.960 | WT *vs.* MMF | *P*=0.062 |
| ***D: Serum protein electrophoresis, Serum parameters*** | | | |  |  |  |  |
| **Fraction 1, Albumin** |  |  |  |  |  |  |  |
|  | WT | 3 | g/dl | 3.019 | 0.259 | **WT *vs.* PLC** | ***P*=0.020** |
|  | PLC | 6 | g/dl | 1.723 | 0.445 | PLC *vs.* MMF | *P*=0.366 |
|  | MMF | 8 | g/dl | 2.103 | 0.533 | **WT *vs.* MMF** | ***P*=0.041** |
| **Fraction 2, Alpha-1** |  |  |  |  |  |  |  |
|  | WT | 3 | g/dl | 0.374 | 0.039 | **WT *vs.* PLC** | ***P*=0.020** |
|  | PLC | 6 | g/dl | 0.114 | 0.048 | PLC *vs.* MMF | *P*=0.398 |
|  | MMF | 8 | g/dl | 0.146 | 0.068 | **WT *vs.* MMF** | ***P*=0.013** |
| **Fraction 3, Alpha 2** |  |  |  |  |  |  |  |
|  | WT | 3 | g/dl | 0.480 | 0.044 | **WT *vs.* PLC** | ***P=*0.028** |
|  | PLC | 6 | g/dl | 0.932 | 0.304 | PLC *vs.* MMF | *P=*0.846 |
|  | MMF | 8 | g/dl | 0.945 | 0.284 | **WT *vs.* MMF** | ***P=*0.041** |
| **Fraction 4, Beta** |  |  |  |  |  |  |  |
|  | WT | 3 | g/dl | 0.532 | 0.134 | **WT *vs.* PLC** | ***P*=0.020** |
|  | PLC | 6 | g/dl | 2.176 | 1.057 | PLC *vs.* MMF | *P*=0.796 |
|  | MMF | 8 | g/dl | 1.953 | 1.232 | WT *vs.* MMF | *P*=0.102 |
| **Fraction 5, Gamma** |  |  |  |  |  |  |  |
|  | WT | 3 | g/dl | 0.895 | 0.068 | WT v*s.* PLC | *P*=1.000 |
|  | PLC | 6 | g/dl | 0.938 | 0.487 | PLC *vs.* MMF | *P*=0.518 |
|  | MMF | 8 | g/dl | 0.641 | 0.329 | WT *vs.* MMF | *P*=0.124 |
| ***E: Urine Dipstick, Semi-quantitative analysis*** | | | |  |  |  |  |
| **Glucose** |  |  |  |  |  |  |  |
|  | WT | 1 | mg/dl | <50 |  |  |  |
|  | PLC | 3 | mg/dl | <50 |  |  |  |
|  |  | 1 | mg/dl | 100 |  |  |  |
|  | MMF | 7 | mg/dl | <50 |  |  |  |
| **Protein** |  |  |  |  |  |  |  |
|  | WT | 1 | mg/dl | <15 | 0 |  |  |
|  | PLC | 4 | mg/dl | 100 | 0 |  |  |
|  | MMF | 8 | mg/dl | 162 | 92 |  |  |
| **Bilirubin** |  |  |  |  |  |  |  |
|  | WT | 1 | mg/dl | 0 | 0 |  |  |
|  | PLC | 4 | mg/dl | 0 | 0 |  |  |
|  | MMF | 7 | mg/dl | 0 | 0 |  |  |
| **Urobilinogen** |  |  |  |  |  |  |  |
|  | WT | 1 | mg/dl | 0 | 0 |  |  |
|  | PLC | 4 | mg/dl | 0 | 0 |  |  |
|  | MMF | 6 | mg/dl | 0 | 0 |  |  |
| **pH** | WT | 1 |  | 5.50 | 0 |  |  |
|  | PLC | 4 |  | 5.75 | 0.29 |  |  |
|  | MMF | 8 |  | 5.63 | 0.23 |  |  |
| **Specific Gravity** |  |  |  |  |  |  |  |
|  | WT | 1 |  | 1.030 | 0 |  |  |
|  | PLC | 5 |  | 1.025 | 0.011 |  |  |
|  | MMF | 8 |  | 1.030 | 0 |  |  |
| **Hemoglobin** |  |  |  |  |  |  |  |
|  | WT | 1 | mg/dl | 0 | 0 |  |  |
|  | PLC | 5 | mg/dl | 1 | 0 |  |  |
|  | MMF | 8 | mg/dl | 1.25 | 0.46 |  |  |
| **Keton** |  |  |  |  |  |  |  |
|  | WT | 1 | mg/dl | 0 | 0 |  |  |
|  | PLC | 4 | mg/dl | 0 | 0 |  |  |
|  | MMF | 6 | mg/dl | 0 | 0 |  |  |
| **Nitrite (qualitative)** |  |  |  |  |  |  |  |
|  | WT | 1 |  | negative |  |  |  |
|  | PLC | 4 |  | negative |  |  |  |
|  | MMF | 7 |  | negative |  |  |  |
| **Leukocyte esterase** |  |  |  |  |  |  |  |
|  | WT | 1 | cells/µl | 0 | 0 |  |  |
|  | PLC | 5 | cells/µl | 250 | 0 |  |  |
|  | MMF | 7 | cells/µl | 250 | 0 |  |  |
| ***F: Urine sediment, Microscopic analysis*** | | |  |  |  |  |  |
| **Bacteria (qualitative)** | |  |  |  |  |  |  |
|  | WT | 1 |  | positive (+++) |  |  |  |
|  | PLC | 1 |  | positive |  |  |  |
|  | MMF | 1 |  | positive |  |  |  |
| **Yeast (qualitative)** |  |  |  |  |  |  |  |
|  | WT | 1 |  | positive (+) |  |  |  |
|  | PLC | 1 |  | positive |  |  |  |
|  | MMF | 1 |  | negative |  |  |  |
| **Red blood cells** |  |  |  |  |  |  |  |
|  | WT | 1 | cells/field | negative |  |  |  |
|  | PLC | 1 | cells/field | 10-20 |  |  |  |
|  | MMF | 1 | cells/field | 5-9 |  |  |  |
| **White bood cells** |  |  |  |  |  |  |  |
|  | WT | 1 | cells/field | 1-4 |  |  |  |
|  | PLC | 1 | cells/field | negative |  |  |  |
|  | MMF | 1 | cells/field | 1-4 |  |  |  |
| **Amorphous Phosphates (qualitative)** | | |  |  |  |  |  |
|  | WT | 1 |  | positive (+) |  |  |  |
|  | PLC | 1 |  | positive |  |  |  |
|  | MMF | 1 |  | positive |  |  |  |
| **Hyaline Cylinders (qualitative)** | | |  |  |  |  |  |
|  | WT | 1 |  | positive (+) |  |  |  |
|  | PLC | 1 |  | positive |  |  |  |
|  | MMF | 1 |  | negative |  |  |  |
| ***G) Differentially phosphorylated proteins in fibrotic kidneys, reversed by mycophonolate mofetil treatment*** | | | | | | |  |
| **Phosphospot 1** |  |  |  |  |  |  |  |
|  | WT | 3 | % | 0.124 | 0.037 | **WT *vs.* PLC** | ***P*<0.050** |
|  | PLC | 3 | % | 0.051 | 0.017 | **PLC *vs.* MMF** | ***P*<0.050** |
|  | MMF | 3 | % | 0.193 | 0.073 | WT *vs.* MMF | *P*=0.275 |
| **Phosphospot 2** |  |  |  |  |  |  |  |
|  | WT | 3 | % | 0.057 | 0.014 | **WT *vs.* PLC** | ***P*<0.050** |
|  | PLC | 3 | % | 0.147 | 0.079 | **PLC *vs.* MMF** | ***P*<0.050** |
|  | MMF | 3 | % | 0.048 | 0.013 | WT *vs.* MMF | *P*=0.275 |
| **Phosphospot 3** |  |  |  |  |  |  |  |
|  | WT | 3 | % | 0.048 | 0.015 | **WT *vs.* PLC** | ***P*<0.050** |
|  | PLC | 3 | % | 0.086 | 0.022 | **PLC *vs.* MMF** | ***P*<0.050** |
|  | MMF | 3 | % | 0.044 | 0.012 | WT *vs.* MMF | *P*=0.827 |
| **Phosphospot 4** |  |  |  |  |  |  |  |
|  | WT | 3 | % | 0.030 | 0.007 | **WT *vs.* PLC** | ***P*<0.050** |
|  | PLC | 3 | % | 0.080 | 0.007 | **PLC *vs.* MMF** | ***P*<0.050** |
|  | MMF | 3 | % | 0.036 | 0.027 | WT *vs.* MMF | *P*=0.827 |
| **Phosphospot 5** |  |  |  |  |  |  |  |
|  | WT | 3 | % | 0.096 | 0.027 | **WT *vs.* PLC** | ***P*<0.050** |
|  | PLC | 3 | % | 0.040 | 0.026 | **PLC *vs.* MMF** | ***P*<0.050** |
|  | MMF | 3 | % | 0.244 | 0.106 | WT *vs.* MMF | *P*=0.127 |
| **Phosphospot 6** |  |  |  |  |  |  |  |
|  | WT | 3 | % | 0.066 | 0.010 | **WT *vs.* PLC** | ***P*<0.050** |
|  | PLC | 3 | % | 0.031 | 0.009 | **PLC *vs.* MMF** | ***P*<0.050** |
|  | MMF | 3 | % | 0.053 | 0.023 | WT *vs.* MMF | *P*=0.513 |
| N: number of mice studied | |  |  |  |  |  |  |
| *P*-value: according to Mann-Whitney test; value in bold print if significant at *P*<0.05 | | | | | |  |  |
| WT: wild-type 129/SvJ mice | |  |  |  |  |  |  |
| PLC: placebo COL4A3-/- mice | | |  |  |  |  |  |
| MMF: COL4A3-/- mice treated with 100 mg/kg mycophenolate mofetil per day | | | | | |  |  |
| *vs.*: *versus* |  |  |  |  |  |  |  |
| ^&^: value under lower limit without dilution | | | |  |  |  |  |
| ^§^: value under lower limit for 1:5 diluted samples | | | |  |  |  |  |
