# Supplementary figures and images for: Effects of mycophenolate mofetil on kidney function and phosphorylation status of renal proteins in Alport COL4A3-deficient mice
Source: Proteome Sci. 2014 Dec 10;12:56. doi: 10.1186/s12953-014-0056-z (PMC4269973; doi:10.1186/s12953-014-0056-z)

## Slide 1
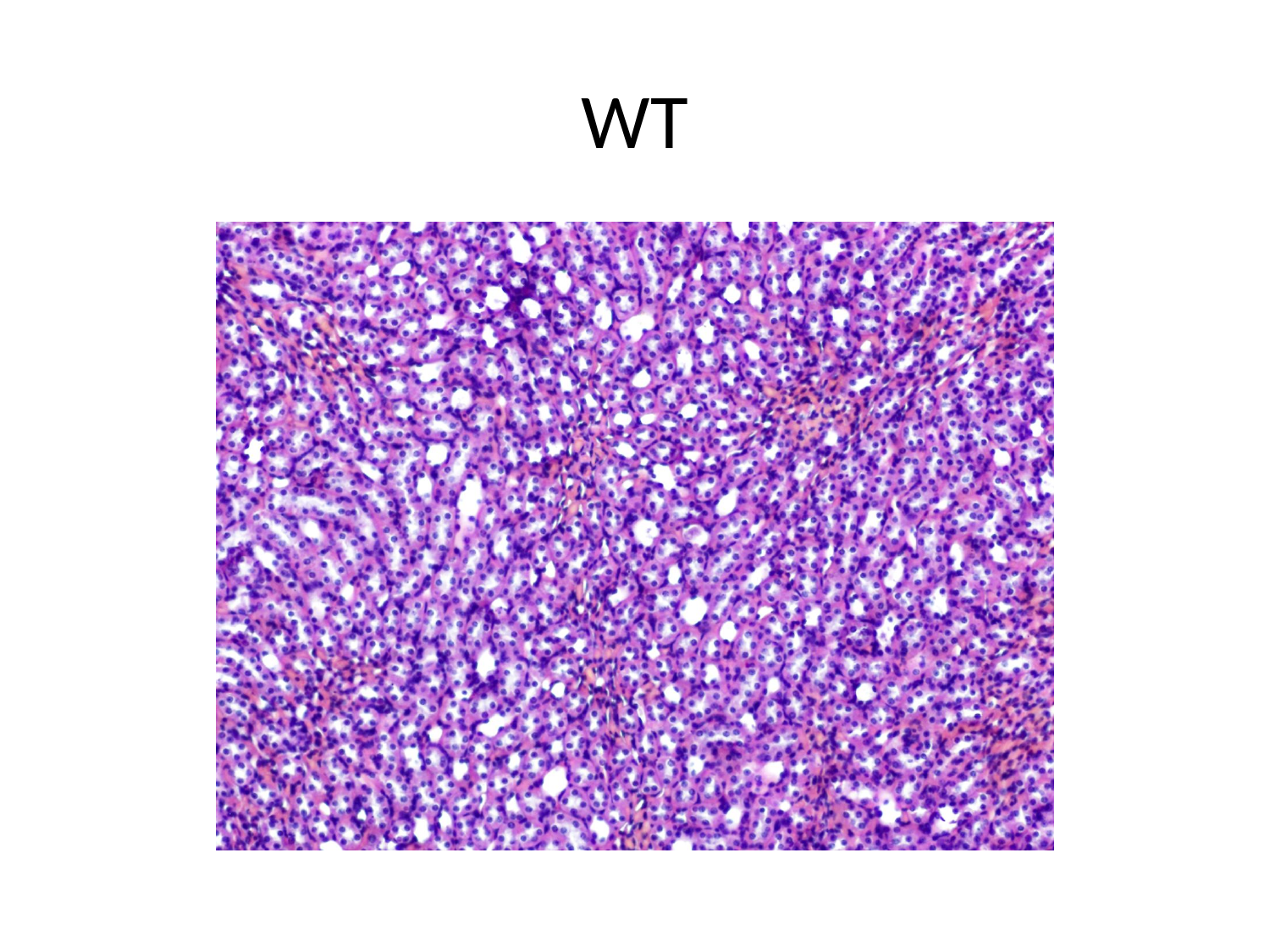

# WT

## Slide 2
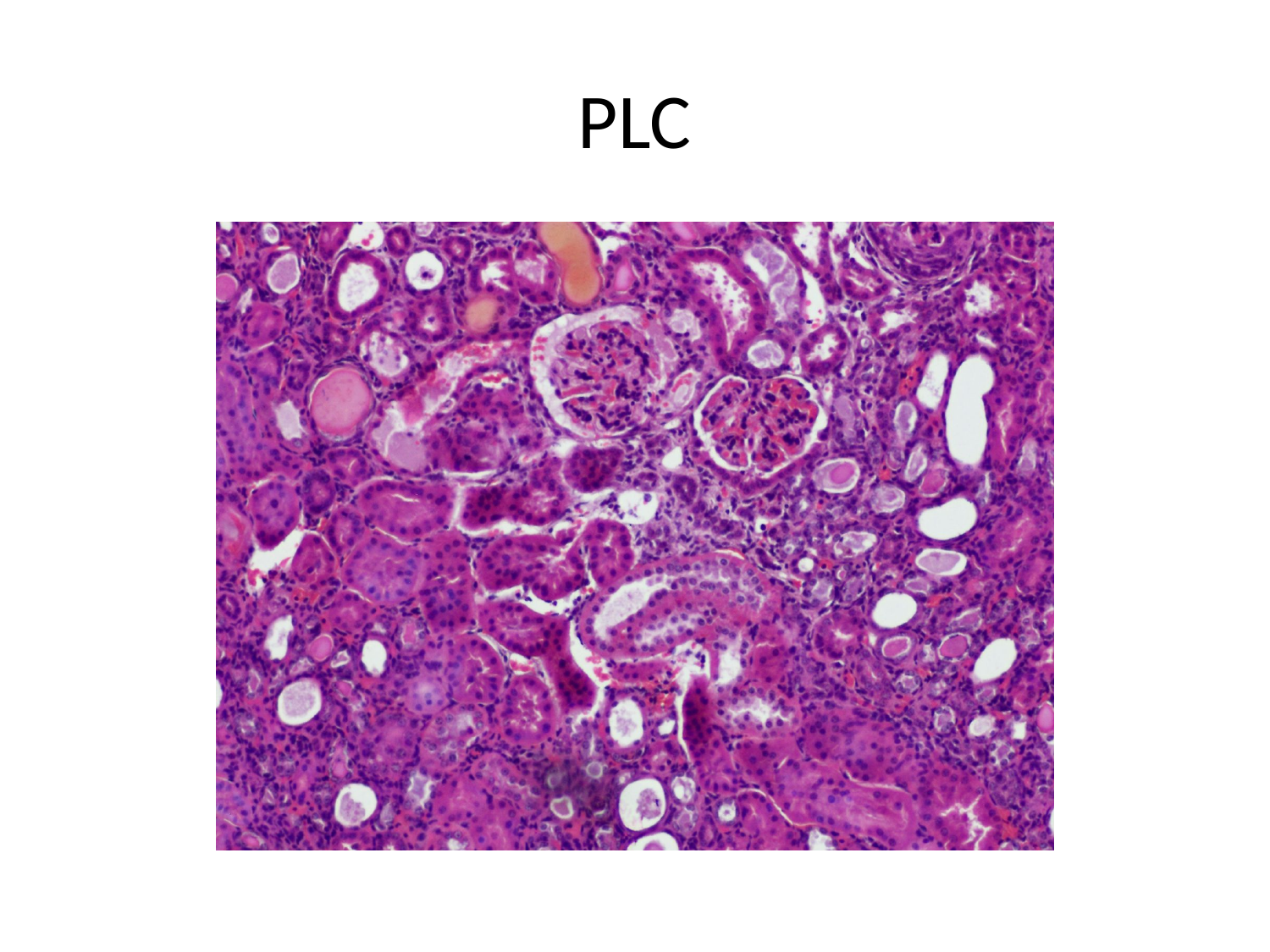

# PLC

## Slide 3
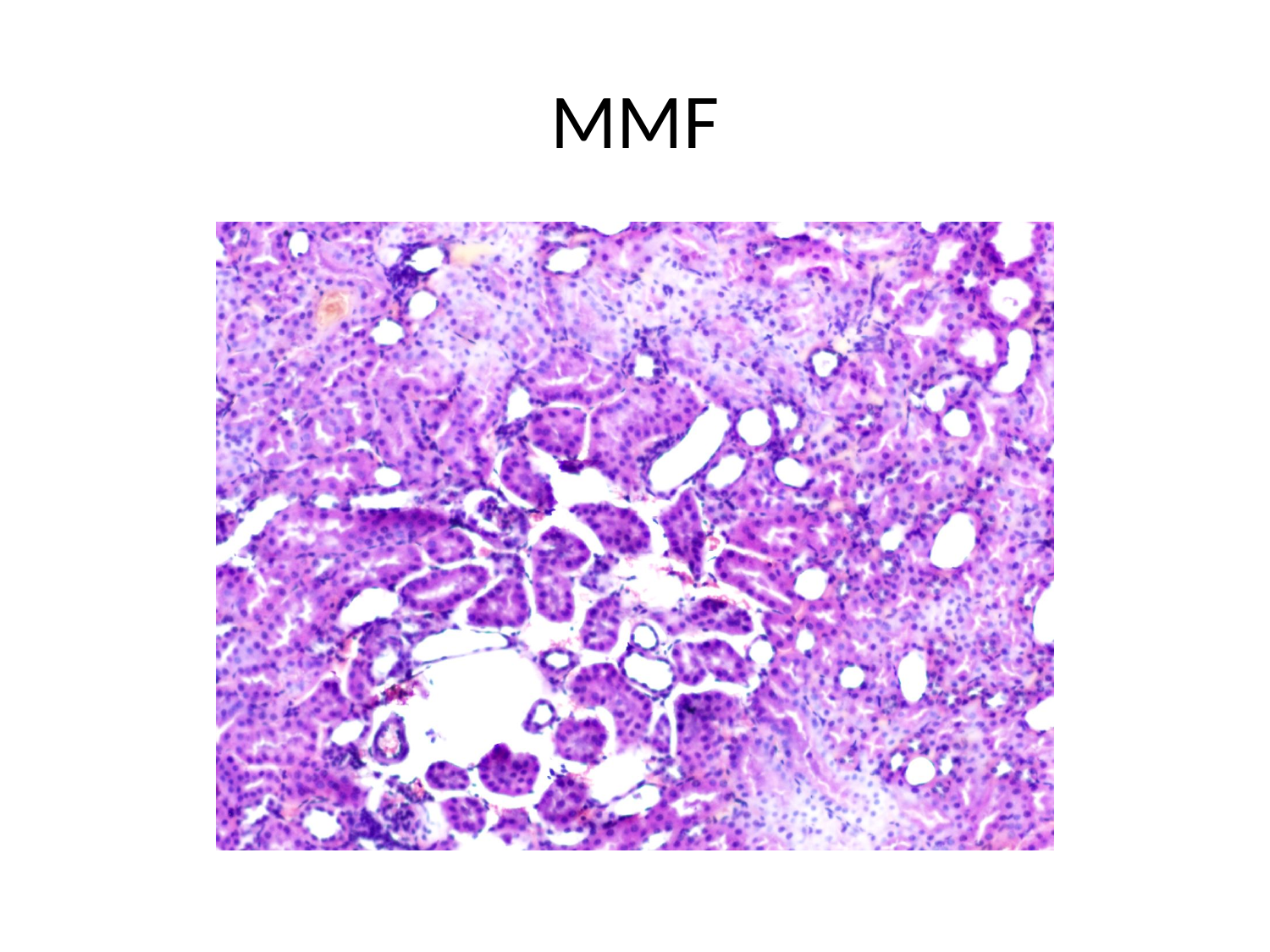

# MMF

Supplement: Additional file 2: Figure S1. — H & E staining. The pictures illustrate the histochemical data from one representative wild-type 129/SvJ mouse (WT); one placebo treated COL4A3−/− mouse (PLC); and one COL4A3−/− mouse treated with 100 mg/kg mycophenolate mofetil per day (MMF). [file 12953_2014_56_MOESM2_ESM.pptx]
