# Supplementary material for: Effects of mycophenolate mofetil on kidney function and phosphorylation status of renal proteins in Alport COL4A3-deficient mice
Source: Proteome Sci. 2014 Dec 10;12:56. doi: 10.1186/s12953-014-0056-z (PMC4269973; doi:10.1186/s12953-014-0056-z)
Supplement: Additional file 3: Table S2. — Protein Overview. The matrix presents an unfiltered list of all proteins identified from the total unique peptides (more than 3) in the six phosphospots of interest. The protein identification presented here was done in one representative wild-type mouse. The main spot protein (printed in bold) was the same as in the PLC and MMF COL4A3−/− mice. [file 12953_2014_56_MOESM3_ESM.docx]

|  |  |  | **Molecular Weight** | **Isoelectric Point** | **Known** | **Phosphospot 1** | **Phosphospot 2** | **Phosphospot 3** | **Phosphospot 4** | **Phosphospot 5** | **Phosphospot 6** |
| --- | --- | --- | --- | --- | --- | --- | --- | --- | --- | --- | --- |
| **#** | **Identified Proteins** | **Entry Name** | **Calculated kDa** | **Basal p*I*** | **Phosphoprotein^a^** | 17 kDa/9 p*I*, observed | 75 kDa/5.5 p*I*, observed | 45 kDa/7.5 p*I*, observed | 27 kDa/6.5 p*I*, observed | 15 kDa/8.5 p*I*, observed | 26 kDa/7 p*I*, observed |
| 1 | Cluster of Keratin, type II cytoskeletal 2 epidermal OS=Mus musculus GN=Krt2 PE=1 SV=1 | K22E_MOUSE | 71 | 8.26 | YES | 26 | 32 | 20 | 16 | 8 | 15 |
| 2 | Glutamate dehydrogenase 1, mitochondrial OS=Mus musculus GN=Glud1 PE=1 SV=1 | DHE3_MOUSE | 61 | 8.05 | YES | 0 | 0 | **20^c^** | 2 | 0 | 0 |
| 3 | ATP synthase subunit alpha, mitochondrial OS=Mus musculus GN=Atp5a1 PE=1 SV=1 | ATPA_MOUSE | 60 | 9.22 | YES | 0 | 0 | 13 | 2 | 0 | 0 |
| 4 | Beta-hexosaminidase subunit beta OS=Mus musculus GN=Hexb PE=2 SV=2 | HEXB_MOUSE | 61 | 8.28 | NO | 0 | 0 | 11 | 0 | 0 | 0 |
| 5 | Lipoamide acyltransferase component of branched-chain alpha-keto acid dehydrogenase complex, mitochondrial OS=Mus musculus GN=Dbt PE=2 SV=2 | ODB2_MOUSE | 53 | 8.78 | NO | 0 | 0 | 10 | 0 | 0 | 0 |
| 6 | Aldehyde dehydrogenase, mitochondrial OS=Mus musculus GN=Aldh2 PE=1 SV=1 | ALDH2_MOUSE | 57 | 7.53 | NO | 0 | 0 | 8 | 0 | 0 | 2 |
| 7 | Cluster of Putative adenosylhomocysteinase 3 OS=Mus musculus GN=Ahcyl2 PE=1 SV=1 (SAHH3_MOUSE) | SAHH3_MOUSE | 67 | 7.13 | YES | 0 | 0 | 8 | 0 | 0 | 0 |
| 8 | Peroxisomal NADH pyrophosphatase NUDT12 OS=Mus musculus GN=Nudt12 PE=2 SV=1 | NUD12_MOUSE | 52 | 6.69 | NO | 0 | 0 | 7 | 0 | 0 | 0 |
| 9 | Propionyl-CoA carboxylase beta chain, mitochondrial OS=Mus musculus GN=Pccb PE=1 SV=2 | PCCB_MOUSE | 58 | 7.59 | NO | 0 | 0 | 7 | 0 | 0 | 0 |
| 10 | Cluster of Keratin, type I cytoskeletal 10 OS=Mus musculus GN=Krt10 PE=1 SV=3 (K1C10_MOUSE) | K1C10_MOUSE | 58 | 5.04 | YES | 6 | 8 | 6 | 7 | 6 | 8 |
| 11 | Peroxisomal acyl-coenzyme A oxidase 1 OS=Mus musculus GN=Acox1 PE=1 SV=5 | ACOX1_MOUSE | 75 | 8.64 | YES | 0 | 0 | 5 | 0 | 0 | 0 |
| 12 | FAD-dependent oxidoreductase domain-containing protein 1 OS=Mus musculus GN=Foxred1 PE=2 SV=1 | FXRD1_MOUSE | 54 | 7.17 | NO | 0 | 0 | 5 | 0 | 0 | 0 |
| 13 | Septin-11 OS=Mus musculus GN=Sept11 PE=1 SV=4 | SEP11_MOUSE | 50 | 6.24 | NO | 0 | 0 | 5 | 0 | 0 | 0 |
| 14 | Gamma-glutamyltranspeptidase 1 OS=Mus musculus GN=Ggt1 PE=1 SV=1 | GGT1_MOUSE | 62 | 6.69 | NO | 5 | 1 | 4 | 0 | 0 | 0 |
| 15 | ATP synthase subunit beta, mitochondrial OS=Mus musculus GN=Atp5b PE=1 SV=2 | ATPB_MOUSE | 56 | 5.19 | YES | 0 | 0 | 4 | 0 | 0 | 0 |
| 16 | Cytochrome P450 4B1 OS=Mus musculus GN=Cyp4b1 PE=1 SV=1 | CP4B1_MOUSE | 59 | 8.67 | NO | 0 | 0 | 4 | 0 | 0 | 0 |
| 17 | 4-trimethylaminobutyraldehyde dehydrogenase OS=Mus musculus GN=Aldh9a1 PE=1 SV=1 | AL9A1_MOUSE | 54 | 6.63 | NO | 0 | 0 | 4 | 0 | 0 | 0 |
| 18 | Catalase OS=Mus musculus GN=Cat PE=1 SV=4 | CATA_MOUSE | 60 | 7.72 | YES | 0 | 0 | 4 | 0 | 0 | 0 |
| 19 | Serine hydroxymethyltransferase, cytosolic OS=Mus musculus GN=Shmt1 PE=1 SV=3 | GLYC_MOUSE | 53 | 6.47 | NO | 0 | 0 | 4 | 0 | 0 | 0 |
| 20 | Long-chain specific acyl-CoA dehydrogenase, mitochondrial OS=Mus musculus GN=Acadl PE=2 SV=2 | ACADL_MOUSE | 48 | 8.53 | NO | 0 | 0 | 3 | 0 | 0 | 0 |
| 21 | Probable aminopeptidase NPEPL1 OS=Mus musculus GN=Npepl1 PE=2 SV=1 | PEPL1_MOUSE | 56 | 6.38 | NO | 0 | 0 | 3 | 0 | 0 | 0 |
| 22 | Histone H4 OS=Mus musculus GN=Hist1h4a PE=1 SV=2 | H4_MOUSE | 11 | 11.36 | YES | 0 | 0 | 3 | 0 | 0 | 0 |
| 23 | Keratin, type I cytoskeletal 42 OS=Mus musculus GN=Krt42 PE=1 SV=1 | K1C42_MOUSE | 50 | 5.09 | NO | 3 | 5 | 3 | 2 | 1 | 2 |
| 24 | Keratin, type I cytoskeletal 16 OS=Mus musculus GN=Krt16 PE=1 SV=3 | K1C16_MOUSE | 52 | 5.13 | NO | 4 | 3 | 3 | 2 | 1 | 1 |
| 25 | Keratin, type I cytoskeletal 17 OS=Mus musculus GN=Krt17 PE=1 SV=3 | K1C17_MOUSE | 48 | 5 | YES | 7 | 3 | 3 | 3 | 1 | 2 |
| 26 | NADH dehydrogenase [ubiquinone] 1 alpha subcomplex subunit 8 OS=Mus musculus GN=Ndufa8 PE=1 SV=3 | NDUA8_MOUSE | 20 | 8.76 | NO | **10^c^** | 1 | 1 | 0 | 0 | 0 |
| 27 | Cluster of Keratin, type II cuticular Hb1 (Fragment) OS=Mus musculus GN=Krt81 PE=2 SV=1 (KRT81_MOUSE) | KRT81_MOUSE | 44 | 5.63 | NO | 0 | 6 | 1 | 0 | 0 | 0 |
| 28 | Dihydrolipoyllysine-residue acetyltransferase component of pyruvate dehydrogenase complex, mitochondrial OS=Mus musculus GN=Dlat PE=1 SV=2 | ODP2_MOUSE | 68 | 8.81 | NO | 0 | 8 | 0 | 0 | 0 | 0 |
| 29 | Estradiol 17-beta-dehydrogenase 8 OS=Mus musculus GN=Hsd17b8 PE=1 SV=2 | DHB8_MOUSE | 27 | 6.1 | NO | 0 | 0 | 0 | **7^c^** | 0 | 3 |
| 30 | Omega-amidase NIT2 OS=Mus musculus GN=Nit2 PE=1 SV=1 | NIT2_MOUSE | 31 | 6.44 | NO | 0 | 0 | 0 | 5 | 0 | **18^c^** |
| 31 | Cathepsin D OS=Mus musculus GN=Ctsd PE=1 SV=1 | CATD_MOUSE | 45 | 6.71 | NO | 0 | 0 | 0 | 4 | 0 | 0 |
| 32 | Serine/arginine-rich splicing factor 1 OS=Mus musculus GN=Srsf1 PE=1 SV=3 | SRSF1_MOUSE | 28 | 10.37 | YES | 0 | 0 | 0 | 3 | 0 | 9 |
| 33 | 26S proteasome non-ATPase regulatory subunit 9 OS=Mus musculus GN=Psmd9 PE=1 SV=1 | PSMD9_MOUSE | 25 | 6 | YES | 0 | 0 | 0 | 3 | 0 | 0 |
| 34 | Cytochrome c1, heme protein, mitochondrial OS=Mus musculus GN=Cyc1 PE=1 SV=1 | CY1_MOUSE | 35 | 9.24 | NO | 0 | 0 | 0 | 2 | 0 | 4 |
| 35 | 26S proteasome non-ATPase regulatory subunit 8 OS=Mus musculus GN=Psmd8 PE=1 SV=2 | PSMD8_MOUSE | 40 | 9.61 | NO | 0 | 0 | 0 | 2 | 0 | 3 |
| 36 | Serum albumin OS=Mus musculus GN=Alb PE=1 SV=3 | ALBU_MOUSE | 69 | 5.75 | YES | 0 | **20^c^** | 0 | 1 | 0 | 0 |
| 37 | ADP/ATP translocase 2 OS=Mus musculus GN=Slc25a5 PE=1 SV=3 | ADT2_MOUSE | 33 | 9.74 | NO | 0 | 0 | 0 | 1 | 0 | 3 |
| 38 | Peptidyl-prolyl cis-trans isomerase A OS=Mus musculus GN=Ppia PE=1 SV=2 | PPIA_MOUSE | 18 | 7.73 | YES | 0 | 0 | 0 | 0 | **12^c^** | 1 |
| 39 | Destrin OS=Mus musculus GN=Dstn PE=1 SV=3 | DEST_MOUSE | 19 | 8.14 | YES | 0 | 0 | 0 | 0 | 6 | 1 |
| 40 | Actin-related protein 2/3 complex subunit 3 OS=Mus musculus GN=Arpc3 PE=1 SV=3 | ARPC3_MOUSE | 21 | 8.78 | YES | 5 | 0 | 0 | 0 | 0 | 0 |
| 41 | Stress-70 protein, mitochondrial OS=Mus musculus GN=Hspa9 PE=1 SV=3 | GRP75_MOUSE | 73 | 5.81 | NO | 0 | 10 | 0 | 0 | 0 | 0 |
| 42 | Adseverin OS=Mus musculus GN=Scin PE=1 SV=3 | ADSV_MOUSE | 80 | 5.64 | YES | 0 | 6 | 0 | 0 | 0 | 0 |
| 43 | Dihydrofolate reductase OS=Mus musculus GN=Dhfr PE=1 SV=3 | DYR_MOUSE | 22 | 8.56 | NO | 3 | 0 | 0 | 0 | 0 | 0 |
| 44 | Glycine N-acyltransferase-like protein Keg1 OS=Mus musculus GN=Keg1 PE=1 SV=1 | KEG1_MOUSE | 34 | 8.69 | NO | 0 | 0 | 0 | 0 | 4 | 0 |
| 45 | Endoplasmic reticulum resident protein 29 OS=Mus musculus GN=Erp29 PE=1 SV=2 | ERP29_MOUSE | 29 | 5.9 | NO | 0 | 0 | 0 | 0 | 0 | 4 |
| 46 | Mitochondrial inner membrane protein OS=Mus musculus GN=Immt PE=1 SV=1 | IMMT_MOUSE | 84 | 6.18 | YES | 3 | 2 | 0 | 0 | 0 | 0 |
| 47 | Peroxiredoxin-5, mitochondrial OS=Mus musculus GN=Prdx5 PE=1 SV=2 | PRDX5_MOUSE | 22 | 9.1 | NO | 0 | 0 | 0 | 0 | 4 | 0 |
| 48 | Dihydropteridine reductase OS=Mus musculus GN=Qdpr PE=1 SV=2 | DHPR_MOUSE | 26 | 7.67 | NO | 0 | 0 | 0 | 0 | 0 | 3 |
| 49 | Enoyl-CoA hydratase domain-containing protein 2, mitochondrial OS=Mus musculus GN=Echdc2 PE=2 SV=2 | ECHD2_MOUSE | 32 | 9.03 | NO | 0 | 0 | 0 | 0 | 0 | 3 |
| 50 | Ig mu chain C region secreted form OS=Mus musculus GN=Igh-6 PE=1 SV=2 | IGHM_MOUSE | 50 | 6.21^b^ | NO | 0 | 3 | 0 | 0 | 0 | 0 |
| 51 | Plastin-3 OS=Mus musculus GN=Pls3 PE=1 SV=3 | PLST_MOUSE | 71 | 5.42 | YES | 0 | 3 | 0 | 0 | 0 | 0 |
| 52 | Actin-related protein 2/3 complex subunit 4 OS=Mus musculus GN=Arpc4 PE=1 SV=3 | ARPC4_MOUSE | 20 | 8.53 | NO | 0 | 0 | 0 | 0 | 3 | 1 |
| 53 | 40S ribosomal protein S14 OS=Mus musculus GN=Rps14 PE=2 SV=3 | RS14_MOUSE | 16 | 10.07 | YES | 0 | 0 | 0 | 0 | 3 | 0 |
| 54 | Hydroxyacylglutathione hydrolase, mitochondrial OS=Mus musculus GN=Hagh PE=2 SV=2 | GLO2_MOUSE | 34 | 7.65 | NO | 0 | 0 | 0 | 0 | 0 | 3 |
| 55 | Heat shock cognate 71 kDa protein OS=Mus musculus GN=Hspa8 PE=1 SV=1 | HSP7C_MOUSE | 71 | 5.37^b^ | YES | 0 | 3 | 0 | 0 | 0 | 0 |
| 56 | Nitrilase homolog 1 OS=Mus musculus GN=Nit1 PE=2 SV=2 | NIT1_MOUSE | 36 | 8.21 | NO | 0 | 0 | 0 | 0 | 0 | 3 |
| # number | | | | | | | | | | | |
| keratin molecules are highlighted in gray | | | | | | | | | | | |
| ^a^ http://www.uniprot.org/; January 2014 | | | | | | | | | | | |
| ^b^ information for the human protein, March, 2014 | | | | | | | | | | | |
| ^c^ main protein in the spot, total unique peptide counts in bold print | | | | | | | | | | | |
